# Enhanced antitumor immunity of nanoliposome-encapsulated heat shock protein 70 peptide complex derived from dendritic tumor fusion cells

**DOI:** 10.3892/or.2015.3934

**Published:** 2015-04-27

**Authors:** YUNFEI ZHANG, WEN LUO, YUCAI WANG, JUN CHEN, YUNYAN LIU, YONG ZHANG

**Affiliations:** 1 Center of Orthopaedic Surgery, Orthopaedics Oncology Institute of Chinese PLA, Tangdu Hospital, Fourth Military Medical University, Xi’an, Shaanxi 710038, P.R. China; 2 Department of Ultrasound, Xijing Hospital, Fourth Military Medical University, Xi’an, Shaanxi 710032, P.R. China

**Keywords:** nanoliposomes, heat shock protein, fusion cells, bioavailability

## Abstract

Tumor-derived heat shock proteins peptide complex (HSP.PC-Tu) has been regarded as a promising antitumor agent. However, inadequate immunogenicity and low bioavailability limit the clinical uses of this agent. In a previous study, we first produced an improved HSP70.PC-based vaccine purified from dendritic cell (DC)-tumor fusion cells (HSP70. PC-Fc) which had increased immunogenicity due to enhanced antigenic tumor peptides compared to HSP70.PC-Tu. In order to increase the bioavailability of HSP70.PC-Fc, the peptide complex was encapsulated with nanoliposomes (NL-HSP70. PC-Fc) in this study. After encapsulation, the tumor immunogenicity was observed using various assays. It was demonstrated that the NL-HSP70.PC-Fc has acceptable stability. The *in vivo* antitumor immune response was increased with regard to T-cell activation, CTL response and tumor therapy efficiency compared to that of HSP70.PC-Fc. In addition, it was shown that DC maturation was improved by NL-HSP70.PC-Fc, which added to the antitumor immunity. The results obtained for NL-HSP70.PC-Fc, which improved immunogenicity and increases the bioavailability of HSP70.PC, may represent superior heat shock proteins (HSPs)-based tumor vaccines. Such vaccines deserve further investigation and may provide a preclinical rationale to translate findings into early phase trials for patients with breast tumors.

## Introduction

Numerous preclinical and clinical studies have shown that tumor-derived heat shock protein-peptide complex (HSP.PC-Tu) can induce antitumor immune responses (1–4) and has been regarded as a promising treatment against tumors. However, the effectiveness of the HSP.PC-Tu vaccines needs to be improved (5–10) to make it a viable treatment. Large-scale phase III clinical trials have demonstrated two major disadvantages of HSP.PC-Tu: the immunogenicity of the peptide complex is inadequate, and as a protein or peptide vaccine the bioavailability of HSP.PC is relatively low (11,12). Thus, the development of novel HSP.PC-Tu vaccines with improved immunogenicity and bioavailability is needed.

To enhance immunogenicity, we previously produced an improved HSP70.PC-based vaccine purified from dendritic cell (DC)-tumor fusion cells (HSP70.PC-Fc), which exhibited improved immunogenicity compared with HSP70.PC-Tu. The immunogenicity of HSP70.PC-Fc is associated with increased content of antigenic tumor peptides and other heat shock proteins, such as HSP90 (13). These results suggest an alternative approach to prepare HSP-based vaccines using DC-tumor fusion technology.

To enhance bioavailability, nanoliposomes were used to encapsulate HSP70.PC-Fc (referred to as NL-HSP70.PC) in this study. The nanocarrier-mediated drug delivery system has been widely used to improve the bioavailability and therapeutic activity of drugs. This technique offers a suitable means of delivering drugs with small molecular weights as well as macro-molecules including proteins, peptides and genes by either localized or targeted delivery to the tissue of interest (14,15). Nanoliposomes have been demonstrated to increase the bioavailability of protein and peptide vaccines due to its tumor affinity, delayed release and prolonged activity (16,17).

Following the encapsulation of nanoliposomes of the HSP70.PC-Fc, referred to as NL-HSP70.PC-Fc, the antitumor immunity was studied. It was demonstrated that NL-HSP70.PC-Fc has acceptable physical and chemical stability, improved antitumor immunity with regard to T-cell activation and CTL responses, and improved tumor therapy efficiency *in vivo* as compared to that of HSP70.PC-Fc. It was also found that DC maturation was improved by NL-HSP70. PC-Fc, a factor that may add to its antitumor characteristics. The results of this study on NL-HSP70.PC-Fc may represent a superior HSPs-based tumor vaccine that deserves investigation and broader application due to its enhanced immunogenicity and bioavailability over non-encapsulated vaccines.

## Materials and methods

### Ethics statements

This study and the experimental protocol involved the use of animals. The animal studies were conducted according to relevant national and international guidelines. The study and protocol were approved by the Institutional Review Board for Animal Participants of the Fourth Military Medical University and Tangdu Hospital. The Ethics Committees of the Fourth Military Medical University approved this procedure.

### Animals and cell lines

Female BALB/c mice were obtained from the Laboratory Animal Center of the Fourth Military Medical University (Xi’an, China) and were used at 6 weeks of age. The poorly immunogenic BALB/c mouse-derived 4T1 mammary carcinoma from the American Type Culture Collection (ATCC, manassas, VA, USA) was used. This tumor shares many characteristics with human mammary cancers, making it an excellent animal model. Additionally, this tumor expresses adequate levels of MHC class I molecules, making it a suitable target for CD8^+^ T-cells(18).

### Preparation of NL-HSP70.PC-Fc

#### Generation of DCs and tumor fusion cells

DCs were generated using the method described by Inaba *et al* (19). Briefly, bone marrow cells were selected by lysis of red cells and depletion of lymphocytes. The cells were then cultured in the presence of granulocyte macrophage colony-stimulating factor (GM-CSF) and interleukin-4 (IL-4) at concentrations of 20 and 100 ng/ml (both compounds, Sigma-Aldrich, St. Louis, MO, USA). On the fifth day of culture, the non-adherent cells were collected as DCs. DC-tumor fusion cells were prepared as previously described by Gong *et al* (13) and Zhang *et al* (20). Tumor cells were mixed with DC preparations at a 1:10 ratio and washed in serum-free pre-warmed RPMI-1640 culture medium. The resulting cell pellet was resuspended in 50% PEG solution (molecular mass: 1,450; Sigma-Aldrich). After 3 min at room temperature, the PEG solution was progressively diluted over the following 5 min with pre-warmed serum-free RPMI-1640 medium. After washing with serum-free RPMI-1640, the resulting fused cells were cultured in RPMI-1640 medium with 20 ng/ml GM-CSF for 3 days by which time each DC-tumor fusion cell had become integrated into a single entity that was loosely adherent to the culture dish.

#### Characterization of DC-tumor fusion cells

To identify the fusion cells, laser confocal microscopy and flow cytometry were used as previously described (21). The tumor cells were labeled with the intracellular fluorescent dye carboxyfluorescein diacetate succinimidyl ester (CFSE; Molecular Probes, Eugene, OR, USA). After PEG fusion with DCs and culture, the hybrid cells were detected by staining with PE-conjugated MHC II antibody, an antibody expressed only by DCs. To verify the hybridoma percentage, a two-color flow cytometry was used. In order to identify the fusion cells, laser confocal microscopy was also used to distinguish the hybrid cells from the parent cells with ease.

#### Immunoprecipitation of HSP70.PC from DC-tumor fusion cells and tumor cells

The HSP70. PC was purified by immunoprecipitation with rabbit anti-mouse HSP70 mAb (Abcam, Burlingame, CA, USA) (13). As described by Gong *et al* (13), DC-tumor fusion cells and tumor cells were collected and washed three times with ice cold phosphate-buffered saline (medium, pH 7.4). The cells were incubated in lysis buffer containing a protease inhibitor cocktail purchased from Roche [50 mM Tris-HCl, pH 7.4, 50 mM NaCl, 1% (v/v) Nonidet P-40, 1 mM Nav] on ice for 30 min, then centrifuged at 13,000 rpm for 15 min. The mAb against HSP70 (Abcam) was added to the supernatant at a concentration of 10 *μ*g/ml and the mixture was gently rotated at 4°C overnight. Then, 80 *μ*l protein A/G-Sepharose (1:1; Sigma-Aldrich) was added and incubated at 4°C for an additional 90 min. The protein Sepharose was collected by centrifugation and after extensive washing in lysis buffer, the immunoprecipitates were eluted into high salt medium (500 mM NaCl). The concentration of protein in the eluate was determined for use in animal vaccination.

#### Encapsulation of nanoliposomes

Distearoylphosphatidylcholine (DSPC), cholesterol and polyethylene glycol (average M.W.2000)-derived distearoylphosphatidylethanolamine (PEG-DSPE2000) were purchased from Avanti Polar Lipids (Genzyme, Cambridge, MA, USA). Liposomes were prepared as previously described (22–24). DSPC, cholesterol and PEG-DSPe2000 (molar ratio is 3:2:0.3) were dissolved in 8 ml chloroform. HSP70.PC-Fc (0.1 mg) was added and mixed by means of magnetic stirrer (300 rpm for 15 min at room temperature). The combined lipids were dried under nitrogen gas and resuspended in 0.9% sterile NaCl at 60°C. Following rehydration, the resulting solution was sonicated for 5 min followed by extrusion through polycarbonate membranes (Corning Costar, Cambridge, MA, USA) of pore size 100 *μ*m consecutively using high-pressure extraction equipment at 55°C to make nanoliposomes of a smaller size. The extra-liposomal salt was removed by a Sephadex G-50 column (Pharmacia, Uppsala, Sweden) equilibrated with normal saline. The final concentration of nanoliposomes was estimated using a phosphate assay (25). The morphology of the liposomes obtained was determined using transmission electron microscopy. Encapsulation efficiency (%) was calculated as: actual amount of drug loaded in nanoparticles/theory amount of drug loaded in nanoparticles × 100 (24). In order to evaluate the stability, NL-HSP70.PC-Fc were kept for 1, 15 and 30 days, at 4°C. Encapsulation efficiency was evaluated at these respective time-points as previously described (26).

#### Immunization regime

Thirty BALB/c mice were divided into 5 groups and immunized subcutaneously (s.c.) with 3 *μ*g NL-HSP70.PC-Fc, HSP70.PC-Fc, HSP70.PC-Tu and nanoliposomes alone NL(−) or medium. Each mouse was immunized on days 0 and 7. On day 14, T cells were harvested from the spleens to analyze their responsiveness.

### Antitumor immunity

#### T-cell activation

T-cell activation by NL-HSP70.PC-Fc was investigated using the IFN-γ enzyme-linked immunosorbent spot assays (IFN-γ ELISPOT kit; Diaclone, Besancon, France) as previously described (21). Spleen cells were harvested by separation on a nylon wool column for use as effector cells and 4T1 breast tumor cells were used as stimulator cells. Stimulator (~5×10^5^) and effector (~5×10^5^) cells were plated onto PVDF-bottomed 96 -well plates coated with anti-IFN-γ antibody. After incubation at 37°C for 24 h, the cells were removed and a biotinylated IFN-γ detection antibody was added for 2 h. Free antibody was washed off and the plates were incubated with streptavidin-alkaline phosphatase for 1 h at 37°C. Streptavidin-alkaline phosphatase bound to biotin was detected via BCIP/NBT substrate (Bio-Rad, Hercules, CA, USA). The resulting spots were counted with a stereomicroscope under magnification of 20–40 times power. A medium control and tumor cells without effector cells were included as negative controls.

#### CTL responses

Analysis of 4T1 breast tumor cell cytotoxicity induced by NL-HSP70.PC-Fc was performed with CytoTox 96 Non-Radioactive Cytotoxicity Assay kits (Promega Inc., Madison, WI, USA) according to the manufacturer’s instructions as previously described (21). Spleen cells harvested by separation on a nylon wool column were used as the effector cells in the CTL assays. 4T1 breast tumor target cells were co-cultured with effector T cells for 4 h at effector/target ratios of 1:12.5, 1:25 and 1:50. After centrifugation at 500 × g for 5 min, 50 *μ*l aliquots of supernatant were transferred to fresh 96-well flat-bottom plates, and an equal volume of reconstituted substrate mix was added to each well. The plates were incubated at room temperature for 30 min and protected from light. Stop solution (50 *μ*l) was added and the absorbance values were measured at 492 nm. The percentage of cytotoxicity for each effector/target cell ratio was calculated as: [A (experimental) − A (effector spontaneous) − A (target spontaneous)] × 100/[A (target maximum) − A (target spontaneous)].

#### In vivo tumor treatment experiment

Thirty mice were implanted with 4T1 tumor cells (2×10^5^ cells/mouse) via orthotopic mammary gland surgical implantation under direct vision (27). The mice were randomly divided into 5 groups of 6 mice and were vaccinated on day 7, week 2 (D14), and week 3 (D21). These mice were subsequently immunized with the same regime as the first vaccination. Tumor volumes (length × width^2^ × π/6) were measured for each individual mouse. Primary tumors were measured every 3 or 4 days following tumor implantation using vernier calipers up to day 35.

#### DC maturation assay

T-cell stimulation by NL-HSP70.PC-Fc requires the participation of an APC such as DCs for antigen representation. To present antigens to T-cells, DCs must fully mature and increase the expression of MHC Class II molecules and costimulatory signals at the cell surface which is necessary to trigger T-cell priming. Then, whether NL-HSP70-PC-Fc can stimulate improved DC maturation was assessed. To determine the DC maturation *in vivo*, inguinal lymph nodes were obtained from mice immunized twice with NL-HSP70.PC-Fc or HSP70.PC-Fc. DCs were collected, stained with anti-MHC Class I, MHC Class II, CD86, CD80 and CD40 Abs (all from BioLegend, San Diego, CA, USA), and analyzed by FACS.

#### Statistical analysis

Data were presented as the mean ± SD. One-way ANOVA and LSD t-tests were used to determine the difference within each group for CTL, IFN-γ ELISPOT and treatment assays. DC maturity assays were analyzed by the Student’s t-test. P<0.05 (P<0.05) was considered statistically significant.

## Results

### Characterization of NL-HSP70.PC-Fc

#### Characterization DC-tumor fusion cells

**Figure 1:**
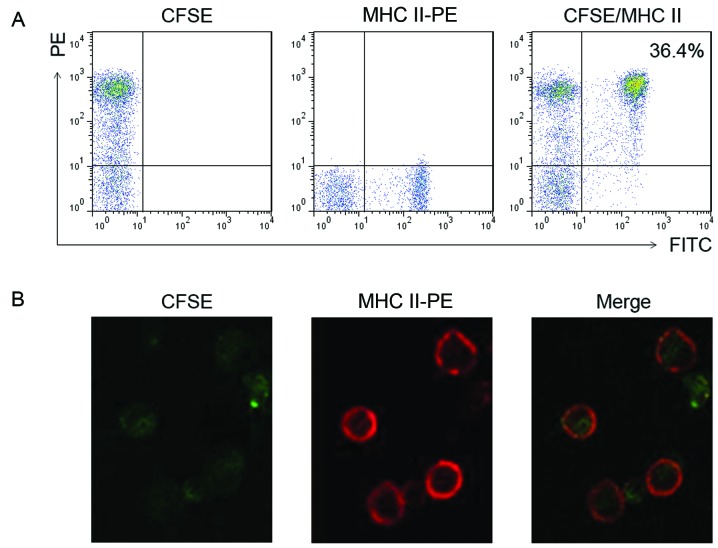
Characterization of DC-tumor fusion cells. (A) Fusion cells were double-stained for the unique dendritic cell marker (MHC Class II-PE) and the tumor marker (pre-stained with CFSE), and analyzed by two-color flow cytometry to quantify the percentage of double-positive fusion cells. (B) Identification of DC-tumor fusion cells using a laser confocal microscope. DC-tumor fusion cells (cytospin on the slides) were identified directly by observation of CFSE-FITC and MHC II-PE under a confocal microscope. Left panel, CFSE-FITC-positive; middle panel, MHC II-PE; right panel, merged image showing double-positive fusion cells. DC, dendritic cells.

The 4T1 tumor cells were pre-labeled with the intracellular fluorescent dye CFSE. DCs expressed high levels of MHC II. After fusion with PEG and 3 days of culture, fusion efficiency was determined by the dual color flow cytometric analysis of fusion cell expression of the DC marker MHC II and the tumor marker CFSE. The fusion efficiency was ~25–58% (Fig. 1a). Successful fusion was confirmed by the observation of individual cells that were positive for CFSE and MHC II by confocal microscopy (Fig. 1B).

#### Characteristics and stability of NL-HSP70.PC-Fc

**Figure 2:**
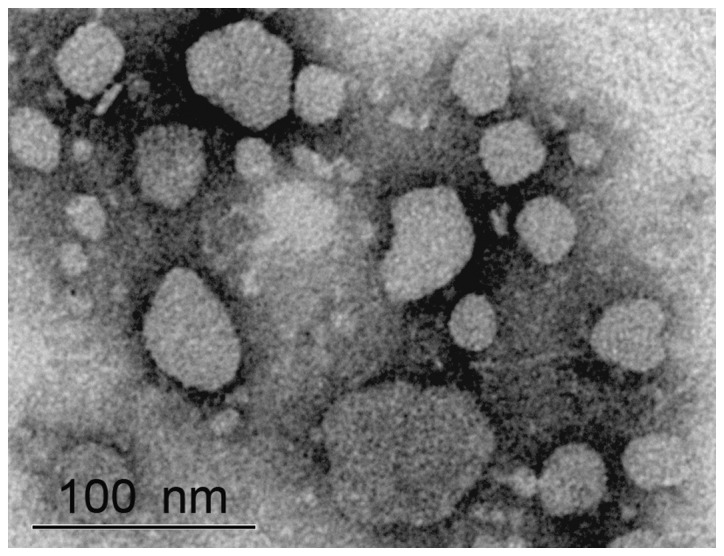
Image of the nanoliposomes encapsulated HSP70.PC-Fc (NL-HSP70.PC-Fc) obtained using a transmission electron microscope (magnification, ×100,000). One drop of diluted NL-HSP70.PC-Fc nanovaccine (1:100) was dropped onto a copper sieve, stained by 0.3% tungsten phosphate, dried and then observed under TEM. The vesicles in nanoliposome were approximately global in shape and the diameter range aws 25–70 nm.

The average diameter of NL-HSP70.PC-Fc was 25–70 nm, as shown in Fig. 2. The encapsulation efficiency was >90%, as calculated by high-performance liquid chromatography and Sephadex^®^ gel filtration. The physicochemical stability of NL-HSP70.PC-Fc is shown in Table I. After 30 days, the encapsulation efficiency had decreased to 91%. Oxidation and hydrolysis of the lipids resulted in the decomposition and aggregation of the liposomes, which also induced drug leakage. PEGylated HSP70.PC-Fc liposomes were stable at low temperatures, although their encapsulation efficiency decreased slightly at 4°C.

#### Enhanced T-cell activation by NL-HSP70.PC-Fc

**Figure 3:**
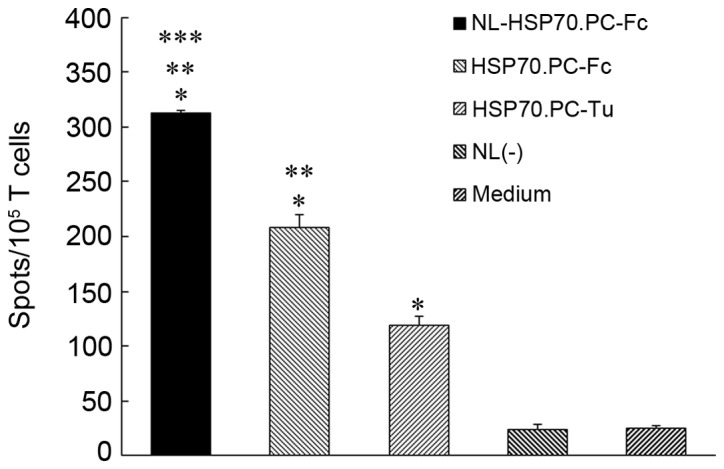
Activation of T-cells against 4T1 breast tumor cells by NL-HSP70.PC-Fc through ELISPOT assay. BALB/c mice were immunized s.c. with NL-HSP70.PC-Fc, HSP70.PC-Fc, HSP70.PC-Tu, NL-(−) or Medium on days 0 and day 7. On day 14, spleen cells were harvested by nylon wool as stimulated T-cells. IFN-γ secretion of T-cells was analyzed using the ELISPOT. The statistical significance was determined by one-way ANOVA (^*^P<0.05, NL-HSP70.PC-Fc/HSP70.PC-Fc/HSP70.PC-Tu vs. medium/NL(−), ^**^P<0.05, HSP70.PC-Fc vs. HSP70.PC-Tu, ^***^P<0.05, NL-HSP70.PC-Fc vs. HSP70.PC-Fc).

To determine T-cell activation induced by NL-HSP70.PC-Fc, IFN-γ production was measured by ELISPOT assays. As shown in Fig. 3, T cells stimulated with NL-HSP70.PC-Fc, HSP70. PC-Fc, or HSP70.PC-Tu produced much more IFN-γ compared to T cells cultured with medium or NL(−). The IFN-γ secretion was much higher in T cells stimulated with NL-HSP70.PC-Fc than those stimulated with HSP70.PC-Fc (P<0.05). IFN-γ secretion was higher in T cells stimulated with HSP70.PC-Fc than those stimulated with HSP70.PC-Tu (P<0.05), which was in line with the previous results.

#### Enhanced CTL activity stimulated by NL-HSP70.PC-Fc

**Figure 4:**
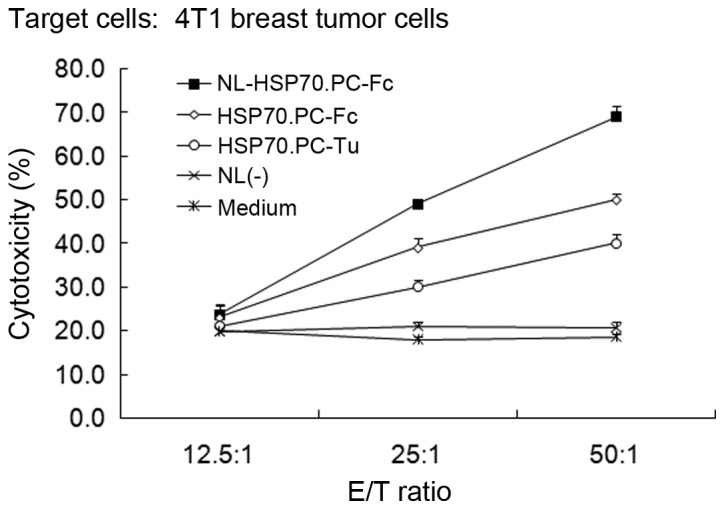
CTL responses against 4T1 breast tumor cells stimulated by NL-HSP70.PC-Fc. BALB/c mice were immunized s.c. with NL-HSP70.PC-Fc, HSP70.PC-Fc, HSP70.PC-Tu, NL(−) or Medium on days 0 and 7. On day 14, spleen cells harvested by separation on a nylon wool column were used as effector cells in CTL assays. 4T1 breast tumor target cells were co-cultured with effector T-cells for 4 h at effector/target cell ratios of 1:12.5, 1:25 and 1:50. All determinations were conducted in triplicate and expressed as the mean value ± SD. The statistical significance was determined by one-way ANOVA. The highest level of lysis was found to be mediated by T-cells stimulated with NL-HSP70.PC-Fc (P<0.05). CTL responses induced by HSP70.PC-Fc was higher than that induced by HSP70.PC-Tu (P<0.05). There were no significant difference in the 1:12.5 group.

The CTL activity against breast tumor cells was determined in LDH release assays. Enhanced CTL activity was observed in T-cells stimulated with NL-HSP70.PC-Fc, HSP70.PC-Fc and HSP70.PC-Tu compared to the control cells (Fig. 4).

The highest level of lysis was found to be mediated by T-cells stimulated with NL-HSP70.PC-Fc (P<0.05). CTL responses induced by HSP70.PC-Fc were higher than that induced by HSP70.PC-Tu (P<0.05).

#### Treatment effect of NL-HSP70.PC-Fc vaccination following tumor implantation

**Figure 5:**
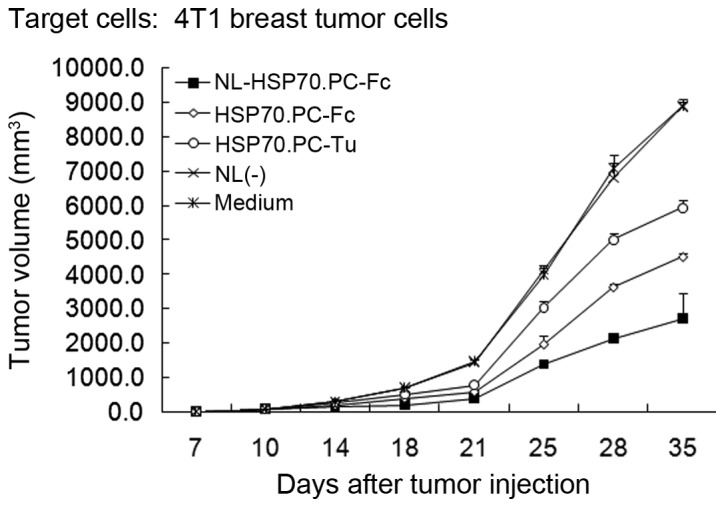
Immunotherapy effect of implanted 4T1 with the tumor vaccine. Mice were s.c. inoculated with 4T1 tumor cells (2×10^5^ cells/mouse respectively and were treated with NL-HSP70.PC-Fc, HSP70.PC-Fc, HSP70.PC-Tu, NL(−) or medium on days 7, 14 and 21. Tumor volumes were measured as described in Materials and methods, and presented as the mean ± SD every 3 or 4 days following tumor challenge using vernier calipers up to day 35. Effective treatment by NL-HSP70.PC-Fc, HSP70.PC-Fc or HSP70.PC-Tu was observed compared with vaccination using NL-(−) or medium. NL-HSP70.PC-Fc significantly delayed tumor growth compared with HSP70.PC-Fc. More effective treatment was induced by HSP70.PC-Fc than that by HSP70.PC-Tu.

Thirty mice were implanted with 4T1 tumor cells and were vaccinated three times according to the immunization regimes respectively. Vaccination with NL-HSP70.PC-Fc, HSP70.PC-Fc or HSP70.PC-Tu significantly delayed tumor growth compared with control using medium or NL(−) (Fig. 5). Compared with HSP70.PC-Fc, the mean tumor volume in the NL-HSP70.PC-Fc group was significantly decreased. Compared with HSP70.PC-Tu, HSP70.PC-Fc significantly delayed tumor growth in the 4T1 tumor model.

#### Enhanced maturation of DCs stimulated by NL-HSP70. PC-Fc

**Figure 6:**
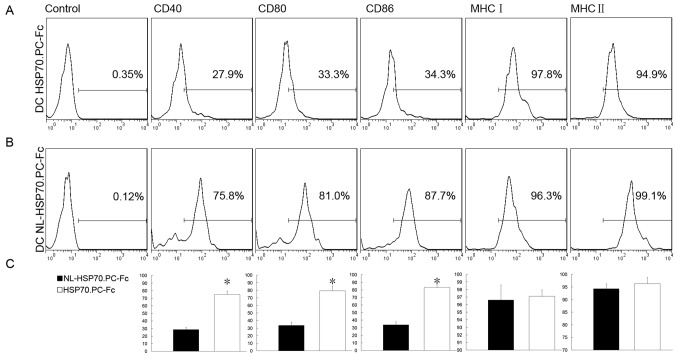
DC maturation stimulated by NL-HSP70.PC-Fc. (A and B) Phenotype of dendritic cells stimulated by HSP70.PC-Fc or NL-HSP70.PC-Fc. Inguinal lymph nodes were obtained from mice immunized twice with NL-HSP70.PC-F or HSP70.PC-Fc. Then dendritic cells were collected, stained with anti-MHC Class I and II, CD86, CD80, CD40 Abs and analyzed by FACS. (C) Quantification of DCs positive for individual mAbs are presented as the mean ± SD. DCs stimulated by NL-HSP70.PC-Fc had significant upregulation of MHC Class II (increased fluorescence intensity) as well as CD80, CD86 and CD40. By contrast, upregulation of these molecules in dendritic cells stimulated with HSP70.PC-Fc was minimal. In each histogram the percentage of positive cells was indicated. DC, dendritic cell.

To stimulate naïve T cells, DCs must become mature, increasing the expression of MHC II molecules and costimulatory signals at the cell surface which is necessary to trigger T-cell priming. As noted in Fig. 6, DCs stimulated by NL-HSP70.PC-Fc showed significant upregulation of MHC Class II expression (increased fluorescence intensity) as well as CD80, CD86 and CD40. By comparison, upregulation of these molecules in DCs stimulated with HSP70.PC-Fc was minimal. A statistical significance in DC maturation stimulated by NL-HSP70.PC-Fc and HSP70.PC-Fc was identified.

## Discussion

The tumor-derived heat shock proteins peptide complex (HSP.PC-Tu) has been regarded as promising treatment against tumors (28,29). Currently, there are more than 150 medical institutions undertaking basic and clinical research on HSPs (30,31). The two largest randomized, open-label, multicenter phase III clinical trials reported in 2008 also confirmed that HSP-based vaccines are safe and clinically feasible (11,12). However, these phase III clinical trials also showed the limitations of HSP-based vaccines. Inadequate immunogenicity and low bioavailability currently restrict the clinical uses of HSP-based vaccine (11,12).

In order to improve the immunogenicity of HSP.PC, we previously created an improved HSP70-based vaccine purified from DC-tumor fusion cells (HSP70.PC-Fc) that optimized the composition of the vaccine. This included increased content of antigenic tumor peptides compared to HSP70.PC-Tu (13). In the current study, it was found that the DC-tumor fusion cell-derived HSP70.PC (HSP70.PC-Fc) increased T-cell activation and CTL response against tumor cells compared to the response induced by HSP70.PC-Tu. These results also support the improved immunogenicity of HSP70.PC-Fc, which is similar to previously performed studies (13).

Although HSP70.PC-Fc has enhanced immunogenicity, as a peptide/protein vaccine, poor bioavailability limits its clinical uses. One major barrier of this limitation is the potential for antigen degradation prior to immune priming. These barriers make delivery of the peptide/protein to antigen-presenting cells (APCs) ineffective (32,33).

The development of nano-based systems has provided protection strategies for incorporated agents, such as biomolecules-nucleic acids, peptides and proteins, which are generally rapidly degraded when administered *in vivo* and through a nano-based delivery system, the bioavailability and pharmacokinetics of tumor vaccine can be improved efficiently (14–16). Berinstein *et al* (34) showed that antigenic peptide-based tumor vaccine encapsulated in nanoliposomes was effective and safe in phase I clinical study. Ge *et al* (35,36) showed that the nanoemulsion-encapsulated maGe1-HSP70/SEA complex protein vaccine markedly enhanced antitumor immunity compared with the vaccine without nano-encapsulation.

PEGylated nanoliposomal technology is a new trend for improving pharmacokinetics, especially for proteins/peptide, because of its many advantages:

i) This technology does not vary the amino acid sequence of a protein and does not cause the covalent attachment of stability factors, unlike other methods such as mutagenesis, direct PEGylation or fusion to an active protein (24,37); ii) it has been shown that proteins/peptides used with this technique remain stable in blood circulation longer than other drugs without this technique (38,39); iii) proteins/peptides encapsulated with this technique have better physiochemical stability than drugs without it (26); iv) PEGylated drugs are water soluble and have a low level of antigenicity and immunogenicity; and v) this technique allows for greater drug specificity and cell targeting (16,40). Although PEGylated nanoliposomes can improve stability, oxidation and hydrolysis of the lipids can result in decomposition and aggregation of the liposomes, which may lead to leakage of inclusions (41,42). Therefore, stability is an important problem for liposomes (43). The formulation strategy is critical for the preparation of nanoliposomes. After preliminary studies to identify the best formulation for PEG liposomes to encapsulate HSP70.PC-Fc, an *in vitro* release study (26) was carried out to evaluate the encapsulation efficiency. The results indicated that PEG-modified HSP70.PC-Fc liposomes had good physicochemical stability, although their encapsulation efficiency decreased slightly at 4°C.

Our study has demonstrated that NL-HSP70.PC-Fc can markedly improve the antitumor immune responses over that of HSP70.PC-Fc. In the T-cell activation assay, IFN-γ secretion was much higher in T cells stimulated with NL-HSP70. PC-Fc than those stimulated with HSP70.PC-Fc. In a CTL assay, the highest level of lysis against tumor cells was found to be mediated by T cells stimulated with NL-HSP70.PC-Fc. In a treatment efficiency assay, vaccination with NL-HSP70.PC-Fc significantly delayed tumor growth as compared to HSP70. PC-Fc and the mean tumor volume in NL-HSP70.PC-Fc group was significantly decreased. These enhanced immune responses may attribute to the enhanced bioavailability of proteins/peptides with encapsulation of nanoliposomes.

The propensity to be phagocytosed efficiently by antigen-presenting cells to induce immune response is critical for antitumor immunity. It was also found that NL-HSP70. PC-Fc can improve DCs maturation as compared to the HSP70. PC-Fc including significant upregulation of MHC Class II (increased fluorescence intensity) as well as CD80, CD86 and CD40. The improved DC maturation by NL-HSP70.PC-Fc may indicate decreased antigen degradation and enhanced immune priming and provide evidence for the increased bioavailability of NL-HSP70.PC-Fc. Our findings that NL-HSP.PC70-Fc may improve maturation of DCs are supported by Im *et al* (44) who showed that nanoliposomes of aspartic acid may enhance the DC maturation

In this study, DC-tumor fusion cell-derived HSP70 peptide complex (HSP70.PC-Fc) tumor vaccine was successfully encapsulated in PEGylated nanoliposomes. It was demonstrated that the NL-HSP.PC70-Fc was uniform in shape, of nanometer-level size, had high stability and maintained encapsulation efficiency. The data presented here shows a clear advantage of the NL-HSP70.PC-Fc as compared to HSP70.PC-Fc: i) NL-HSP70.PC-Fc induced greater T-cell activation; ii) NL-HSP70.PC-Fc elicited more powerful CTL responses; iii) NL-HSP70.PC-Fc induced a more significant treatment effect; and iv) NL-HSP70.PC-Fc stimulated maturation of DCs better than HSP70.PC-Fc.

As a result of this study, the nanoliposome encapsulated HSP70.PC derived from DC-tumor fusion cells (NL-HSP.PC70-Fc) has been found to improve the immunogenicity as compared to HSP.PC70-Tu derived from tumors, and increase the bioavailability compared to HSP.PC70-Fc. These findings may represent superior HSPs-based tumor vaccines which deserve further investigation and broader applications. However, the manner in which NL-HSP70.PC-Fc induced improved bioavailability and what is the detailed mechanisms behind the enhanced antitumor immune responses remain to be elucidated. Exploring the detailed pharmacokinetics of NL-HSP70.PC-Fc is required for further investigation.

## Figures and Tables

**Table I:** Encapsulation efficiency analyzed at different time-points at 4°C.

Time (days)	1	15	30
Encapsulation efficiency (%)	95±2.1	93±0.9	91±1.8

To evaluate the stability, NL-HSP70.PC-Fc were kept for 1, 15 and 30 days at 4°C. Encapsulation efficiency was evaluated at these respective time-points.
